# Specificity of Immunoglobulin Response to Nontuberculous Mycobacteria Infection in People with Cystic Fibrosis

**DOI:** 10.1128/spectrum.01874-22

**Published:** 2022-07-06

**Authors:** Kenneth C. Malcolm, Emily A. Wheeler, Kara Calhoun, Patricia M. Lenhart-Pendergrass, Noel Rysavy, Katie R. Poch, Silvia M. Caceres, Milene T. Saavedra, Jerry A. Nick

**Affiliations:** a Department of Medicine, National Jewish Health, Denver, Colorado, USA; b Department of Medicine, University of Colorado, Aurora, Colorado, USA; c Department of Pediatrics, University of Colorado, Aurora, Colorado, USA; Quest Diagnostics

**Keywords:** ELISA, cystic fibrosis, immunoglobulins, nontuberculous mycobacteria, serodiagnosis

## Abstract

Nontuberculous mycobacteria (NTM) infections are increasingly prevalent in chronic lung diseases, including cystic fibrosis (CF). Mycobacterium abscessus is of particular concern due to relatively greater virulence and intrinsic antimicrobial resistance. Airway culture identification, the standard method for detecting pulmonary infection, is hindered by low sensitivity, long culture times, and reliance on sputum production or lavage. A culture-independent test for detecting NTM infection could complement, or replace, sputum culture, which is becoming more difficult to obtain with reduced sputum production by people with CF (pwCF) on highly effective modulator therapy. We describe an assay for the detection of plasma anti-M. abscessus antibodies of pwCF to antigens from M. abscessus lysates. Anti-M. abscessus IgG and IgA, but not IgM, discriminated with high specificity subjects infected with M. abscessus from those infected by M. avium complex, and from those with distant or no NTM infections. The IgG3 subclass predominated with minor contributions by other subclasses. Both aqueous and organic soluble antigens were recognized by plasma IgG. A validation cohort measuring IgG and IgG3 identified M. abscessus positive subjects, and elevated IgG was sustained over several years. These studies show the benefit of M. abscessus cell lysates to detect plasma IgG of subjects with CF and M. abscessus infections. Subclass analysis suggests that IgG3 is the predominant subtype in these subjects with chronic bacterial infections suggesting a defect in class maturation. Serodiagnosis could be useful to monitor M. abscessus group infections in chronic lung disease as an adjunct or alternative to culture.

**IMPORTANCE** Lung infections with nontuberculous mycobacteria (NTM), and particularly Mycobacterium abscessus, a pathogen with high antibiotic resistance, are of great concern due to poor clinical outcomes and challenging detection in people with cystic fibrosis and other diseases. Standard detection methods are insensitive and increasingly difficult. We describe the measurement of NTM-specific antibodies from plasma to identify subjects infected with M. abscessus. The assay is sensitive and provides information on the immune response to NTM infections. This assay could be used to help identify subjects with NTM pulmonary infections and track disease progression, either alone or in conjunction with other tests.

## INTRODUCTION

Nontuberculous mycobacteria (NTM) are increasingly observed in patients with chronic pulmonary disease such as cystic fibrosis (CF), alpha-1 antitrypsin deficiency, and chronic obstructive pulmonary disease (1, 2). Mycobacterium abscessus (MABSC) infections are recognized as having a particularly poor prognosis due to accelerated decline in lung function, extensive antimicrobial resistance and treatment failure, and diagnostic challenges in distinguishing active disease-causing infection from indolent or transient infection (3). M. avium complex (MAC) infections are also commonly detected and have similar diagnostic and therapeutic challenges.

Microbiological culture of sputum is the only method in common use for detection of pulmonary bacterial infections. However, sputum heterogeneity can lead to inconsistent pathogen detection (4–6). Detection of NTM requires decontamination to eliminate more prolific bacteria, which results in reduced NTM viability. Sensitivity of culture results is further complicated by slow NTM growth. Clinical and genetic data suggest that people with CF (pwCF) can harbor NTM infections for years before detection by culture (7, 8). Furthermore, spontaneous sputum production is often unavailable, particularly in children and in many pwCF following the introduction of highly effective cystic fibrosis transmembrane regulator (CFTR) modulator therapies. Bronchoscopy is a reliable sampling of the airway environment but is invasive and samples only a small portion of the lung. A minimally invasive test independent of sputum production or highly invasive procedures would be useful in detecting infection and to confirm culture results.

One culture-independent strategy for pathogen detection relies on the robust humoral immune response to infection. Induction of plasma antibodies can occur within days of exposure to pathogens and are generally maintained for the duration of infection and for months following pathogen clearance. Detection of Pseudomonas aeruginosa-specific antibodies correlates well with culture results (9–15), and circulating antibody levels have been used to predict the onset of infection and response to treatment in pwCF (9–12, 14).

Antibody responses to NTM infection have been demonstrated, but the clinical and functional significance of antibody production is unclear. NTM infection status has been estimated by measuring anti-MAC antibodies in plasma (16–20). These studies consistently detect samples from culture-positive subjects and discriminate between subjects with and without pulmonary disease and correlate with treatment outcome (16–19). However, these methods poorly discriminated MABSC from MAC infections (19–21), likely due to the use of single, common mycobacterial antigens. Measurement of M. abscessus antibodies has been reported less frequently and fail to distinguish MABSC from MAC infections, as they also used common mycobacterial antigens such as the glycopeptidolipid core or antigen A60 (19–22). A recent publication described antibodies against a protein fraction of rough M. abscessus and recombinant phospholipase C (PLC) with an improved ability to separate MABSC from MAC infections (23). To address these issues, we developed an ELISA to detect plasma anti-M. abscessus antibodies using bacterial lysates to assess M. abscessus infection status and immunoglobulin profile. Levels of specific anti-M. abscessus antibody classes and subclasses correlate well with culture results and have high specificity for MABSC. Specifically, the IgG response predominates and is due largely to IgG3 with a minor contribution of IgG1, suggesting a constrained use of the antibody repertoire. This assay may be useful to rapidly detect infection status in support of sputum culture results or as a culture-independent alternative to monitor subjects for follow-up.

## RESULTS

### Assay development.

**FIG 1:**
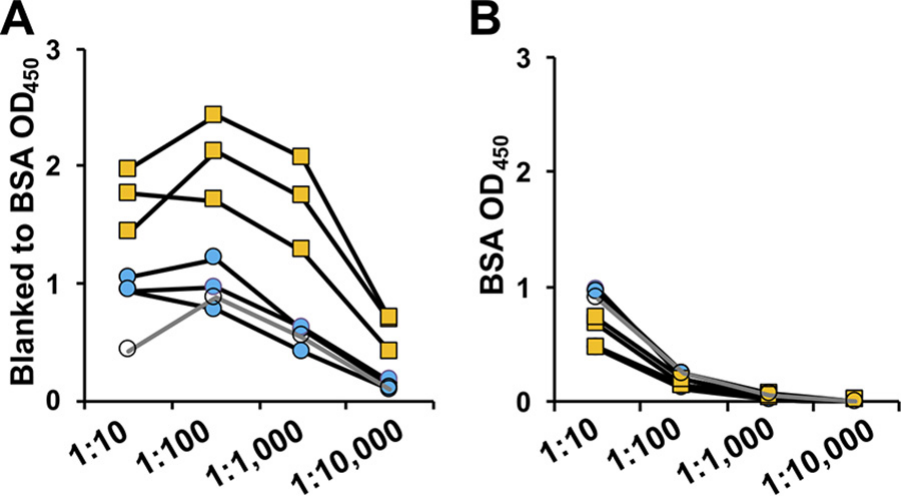
Optimization of dilutions to detect anti-M. abscessus IgG. Serial dilutions of plasma from MABSC+ subjects (orange squares), MAC+ subjects (blue circles), and an NTM-free subject (open) were incubated with wells coated with (A) M. abscessus lysates after blanking to BSA signal or (B) BSA. IgG was detected with HRP-conjugated anti-IgG.

Unfractionated M. abscessus lysates were used to test the humoral response to infection by providing a comprehensive range of M. abscessus antigens, including protein, lipid, carbohydrate, and complex molecular antigens from both extracellular and intracellular compartments. Initial tests determined the plasma dilutions for the specific detection of anti-M. abscessus IgG. Using serial plasma dilutions we assayed three subjects with known recent MABSC infections, two subjects with recent MAC infections, one subject with a distant (~3 years) MAC infection, and one subject with no history of NTM infection. The signal best discriminated between MABSC and MAC at dilutions of 1:1,000 to 1:10,000 (Fig. 1A), and nonspecific binding to bovine serum albumin (BSA) was minimal (Fig. 1B).

### Assessment of IgG, IgA, and IgM levels in plasma of a characterized cohort.

We analyzed 45 plasma samples from pwCF for M. abscessus-specific IgG, IgA, and IgM, the three main classes of plasma immunoglobin. This cohort was characterized for NTM culture status over a 5-year period. The MABSC+ cohort included 15 subjects with MABSC infections. The subspecies identified by cultures were nine M. abscessus subsp. *abscessus*, five M. abscessus subsp. *massiliense*, and one M. abscessus subsp. *bolletii*. Plasma was collected 26.1 ± 32.6 days from last positive culture. The MAC+ cohort consisted of 13 subjects with MAC only infection. The species identified by culture were six M. intracellulare, four M. avium, and three *M. chimaera*. Plasma was collected 340.1 ± 457 days after positive culture. Although a sizeable difference in the time of collection is suggested, the difference between MABSC+ and MAC+ collection days was not significant. A total of 17 subjects with no history of NTM-positive cultures over at least a 5-year period (NTM-free) were also included. Four of the subjects coculturing MABSC and MAC species were included in the MABSC+ cohort. This cohort was previously used to discriminate between lifetime NTM-positive and -negative status based on urine lipoarabinomannan levels (24).

**FIG 2:**
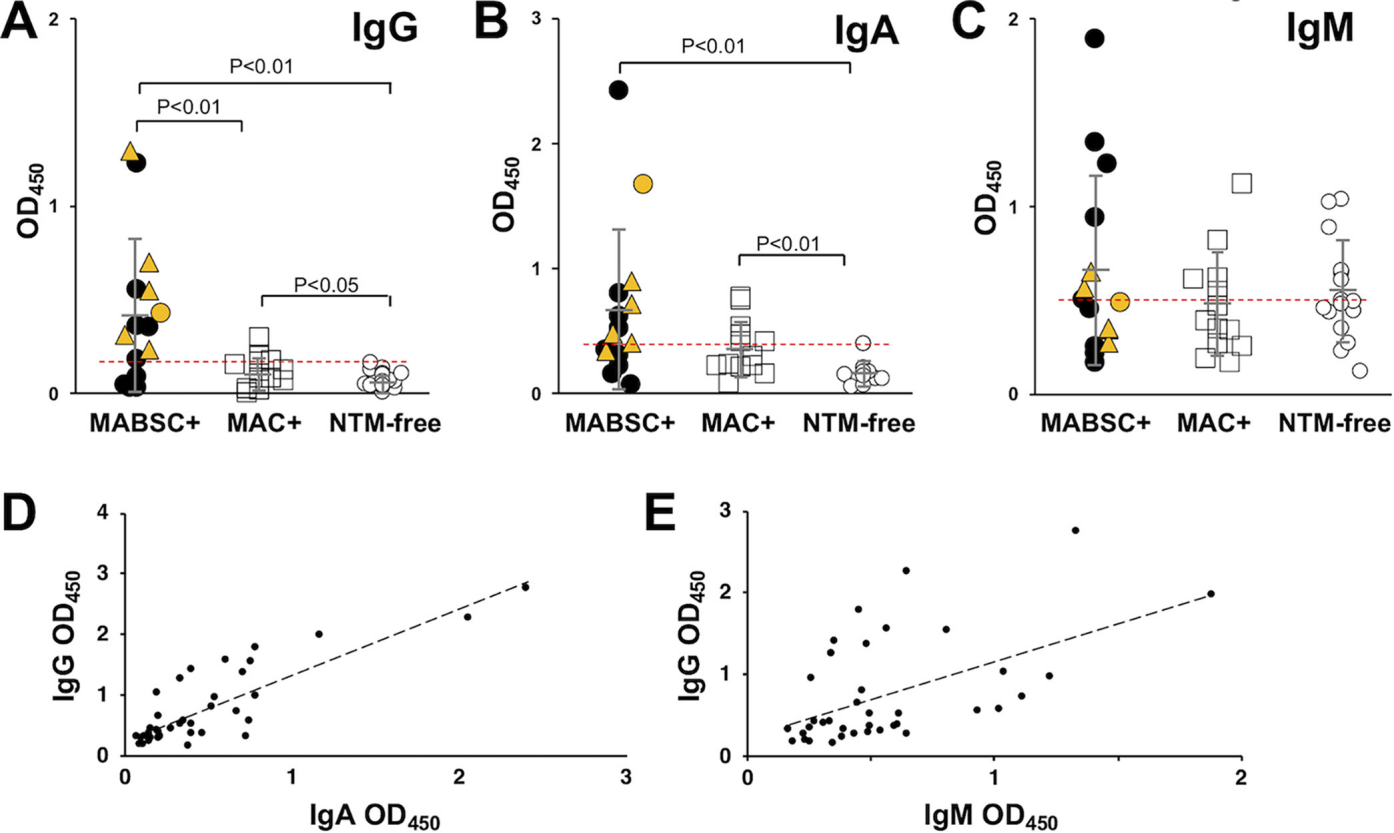
Anti-M. abscessus IgG and IgA are specific for detecting MABSC+ subjects. Detection of anti-M. abscessus IgG (A), IgA (B), and IgM (C) on plates coated with M. abscessus lysates. Correlation of IgG and IgA (D) and IgG and IgM (E) are shown. Red dashed line represents the cut-off for detection determined from the ROC curve. Cut-off values were 0.179 (IgG), Cut-off values were 0.179 (IgG), 0.400 (IgA) and 0.486 (IgM). M. abscessus positive subjects (closed circles), *M. massiliense* (orange triangles), and *M. bolletii* (orange circle); MAC+ (open square); NTM-free (open circle); gray bars indicate mean +/− SD.

We detected anti-M. abscessus IgG, IgA, and IgM in plasma (Fig. 2A to C). For both IgG and IgA, plasma samples from MABSC+ subjects had higher antibody levels than MAC+ and NTM-free samples. IgG levels did not correlate between the time from last positive culture and time of sample collection (Fig. S1). MAC+ subjects had higher IgG and IgA levels compared with the NTM-free group. The difference for IgA between MABSC+ and MAC+ was not significant. In addition, NTM positive samples (MABSC+ and MAC+) had greater IgG and IgA levels than NTM-free samples (*P* < 0.01). IgM levels were not distinguishable between groups. Receiver-operator characteristic (ROC) curve analysis showed areas-under-the-curve (AUROC) for IgG, IgA, and IgM of 0.83 and 0.76, and 0.57, respectively (Table 1). Using the point nearest the upper left corner of the ROC curves as a cut-off determination, the sensitivity and specificity of the MABSC IgG signal were, respectively, 66% and 89%, for IgA 60% and 75%, and for IgM 46% and 55% (Table 1). ROC curves are shown in Fig. S2. Levels of IgG were positively correlated with both IgA (r^2^ = 0.68; *P* = 2.9 × 10^−8^) and IgM (r^2^ = 0.43; *P* = 6.9 × 10^−5^) (Fig. 2D and E). Similar results were obtained when absorbance levels greater than 2 SD above the mean levels of NTM-free subjects served as a cut-off for positive ELISA results (not shown). Assay signal and time from last positive culture was observed (not shown). The coefficients of variation (CV) for IgG and IgA were calculated to be 5.2% and 3.7%, respectively. Too few replicates for IgM were obtained to measure CV%. Descriptive statistics for IgG, IgA, and IgM are shown in Table 1.

**TABLE 1:** Descriptive statistics for detection of MABSC+ by immunoglobulin class

Culture status	Assay result	IgG	IgG1	IgG2	IgG3	IgG4	IgA	IgM
MABSC+	True positive	10	8	5	10	7	9^*a*^	7
	False negative	5	7	10	4	8	6	8
MABSC−	False positive	3	7	9	4	9	7	13
	True negative	26	22	20	25	20	22	16
Sensitivity (%)		66.7	53.3	33.3	71.4	46.7	60.0	46.7
Specificity (%)		89.7	75.9	69.0	86.2	69.0	75.9	55.2
PPV^*b*^ (%)		76.9	53.3	35.7	71.4	43.8	56.3	35.0
NPV^*c*^ (%)		83.9	75.9	66.7	86.2	71.4	78.6	66.7
AUROC^*d*^		0.83	0.68	0.51	0.84	0.62	0.76	0.57

a Number of subjects in each category.

b PPV, positive predictive value.

c NPV, negative predictive value.

d AUROC, Area-under-the-ROC curve.

### Detection of anti-M. abscessus antibodies to aqueous- and organic-soluble antigens.

To further characterize the antibody response, the M. abscessus lysate fractionated with chloroform/methanol to separate aqueous and organic fractions were used as antigens to detect anti-M. abscessus IgG. Both antigen sources distinguished MABSC samples from MAC and NTM-free samples (Fig. S3). The sensitivity and specificity of detecting IgG for MABSC positive subjects were 64% and 71% for the aqueous fraction and 64% and 78% for the organic fraction (Table S1). The AUROCs for the aqueous and organic assays were 0.73 and 0.77, respectively. Signals from both fractions were highly correlated (Fig. S3C; r^2^ = 0.67; *P* = 5 × 10^−8^) indicating that both aqueous- and organic-soluble moieties are antibody targets.

### Detection of plasma IgG subclasses.

**FIG 3:**
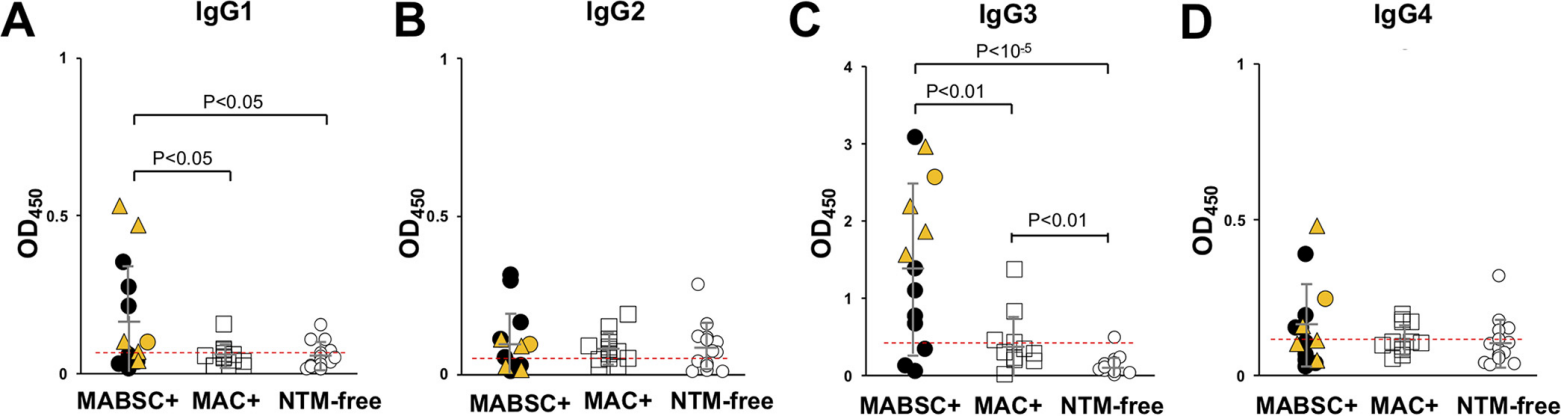
Detection of IgG subclass antibodies to M. abscessus. Detection of (A) IgG1; (B) IgG2; (C) IgG3; (D) IgG4 on plates coated with M. abscessus lysates. Red dashed line represents the cut-off for detection determined from the ROC curve. Cut-off values were 0.065 (IgG1), 0.106 (IgG2), 0.423 (IgG3) and 0.113 (IgG4). M. abscessus positive subjects (closed circles), *M. massiliense* (orange triangles), and *M. bolletii* (orange circle); MAC+ (open square); NTM-free (open circle); gray bars indicate mean +/− SD.

The IgG-detecting antibody recognizes all four IgG subclasses. To establish the IgG subclasses responsible for the elevation of plasma anti-M. abscessus IgG we measured the presence of IgG1, IgG2, IgG3, and IgG4 (Fig. 3). IgG3 gave the highest signal and was significantly higher in MABSC+ than in MAC+ or NTM-free subjects. NTM positive samples had greater IgG3 levels than NTM-free samples (*P* < 0.01). IgG1 was also able to discriminate between MABSC+ from the MAC+ and NTM-free groups, but not MAC+ or NTM+ from NTM-free. Neither IgG2 nor IgG4 were able to detect differences between any groups. The sensitivity and specificity for IgG3 (71% and 86%, respectively) was similar to that of total IgG (66% and 89%), but those of IgG1 (53% and 75%), IgG2 (33% and 69%), and IgG4 (46% and 69%) were lower. The AUROC for IgG1, IgG2, IgG3, and IgG4 were 0.68, 0.51, 0.84, and 0.62, respectively. Descriptive statistics for the four subclasses are depicted in Table 1. The coefficients of variation for IgG1 and IgG3 were calculated as 11.1% and 13.7%, respectively. When combining data for IgG subclasses or of the IgG subclasses with IgA the AUROC was not much improved over that of IgG3 alone (AUROC 0.84); AUROCs for IgG1+IgG3, IgG1+IgA, and IgG3+IgA were 0.86, 0.84, and 0.85, respectively. Unsupervised analysis of all antibody data by hierarchical clustering indicates a close relationship among the MABSC+ samples (Fig. S4) and an enrichment of non-*abscessus* subspecies in the major MABSC+ cluster. An elevated IgG3 signal is apparent with MABSC+ and IgM signal is more associated with MAC+ and NTM-free samples. A mixed subset of samples is highest in IgA.

### Assessment of a validation cohort.

**FIG 4:**
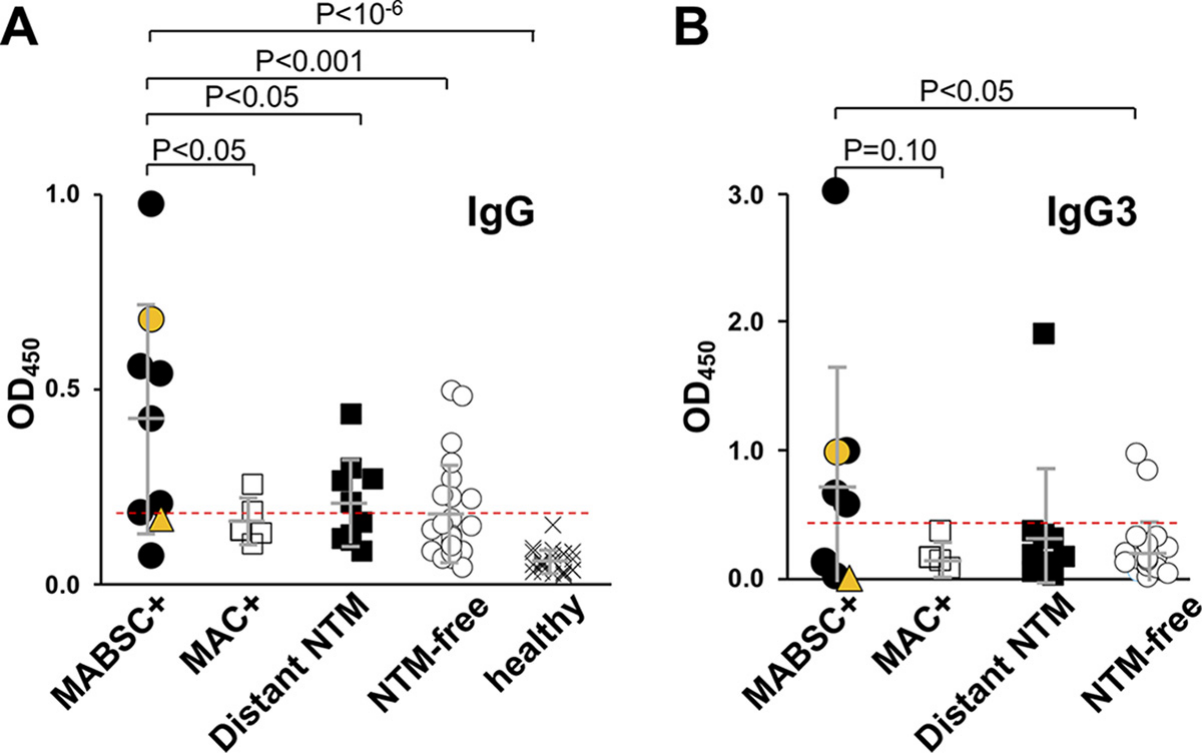
Detection of anti-M. abscessus from an independent cohort of CF subjects. (A) IgG and (B) IgG3 were detected from plasma of MABSC+, MAC+, distant (>3 yrs) positive NTM cultures, NTM-free, and healthy subjects on plates coated with M. abscessus lysates. Cut-offs (red dashed line) were from those in Fig. 2 and 4, respectively. Orange symbols represent subjects with *M. bolletii* (circle) and *M. massiliense* (triangle); gray bars indicate mean +/− SD.

An independent cohort of subjects blinded as to infection status was assayed to validate the IgG and IgG3 results in the training cohort. Unblinding revealed that nine (18.7%) of the 48 subjects in the validation cohort were culture-positive for MABSC within a 36-month window before or after sample collection (seven M. abscessus subsp. *abscessus*; one M. abscessus subsp. *bolletii;* one *M. massiliense*); five (10.4%) were culture-positive for MAC within a 36-month window before or after sample collection (four M. avium, one M. intracellulare); 10 (20.8%) were NTM culture positive more than 36 months before or after sample collection (distant NTM infection) or had nonpathogenic NTM such as M. gordonae; and 24 (50%) had no history of positive NTM culture. Twenty-four healthy donors were also included in this analysis. The window of NTM positivity was derived from clinical and genetic data indicating up to a 3-year delay between NTM acquisition and culture positivity (7, 8). IgG signals were significantly higher in MABSC+ samples than from those with MAC infections, distant NTM infections, NTM-free subjects, and healthy control cohorts (Fig. 4). The same dilutions and cut-off from the training cohort were used. IgG levels did not correlate between the time from last positive culture, either before or after sampling, and time of sample collection (Fig. S5). The specificity and sensitivity of IgG for detection of MABSC+ were 61% and 77%, respectively. For IgG3, there was a trend to a higher signal for MABSC+ subjects than for MAC+ subjects (*P* = 0.10), and a significant difference between MABSC+ and NTM-free subjects. The specificity and sensitivity were 92% and 55%, respectively. Descriptive statistics are presented in Table 2.

**TABLE 2:** Descriptive statistics for detection of MABSC+ in an independent cohort of pwCF

Culture status	Assay result	IgG	IgG3
MABSC+	True positive	7	5
	False negative	2	4
MABSC−	False positive	15	3
	True negative	24	35
Sensitivity (%)		77.8	55.6
Specificity (%)		61.5	92.1
PPV^*a*^ (%)		31.8	62.5
NPV^*b*^ (%)		92.3	89.7

a PPV, positive predictive value.

b NPV, negative predictive value.

### Longitudinal detection of anti-MABSC IgG.

**FIG 5:**
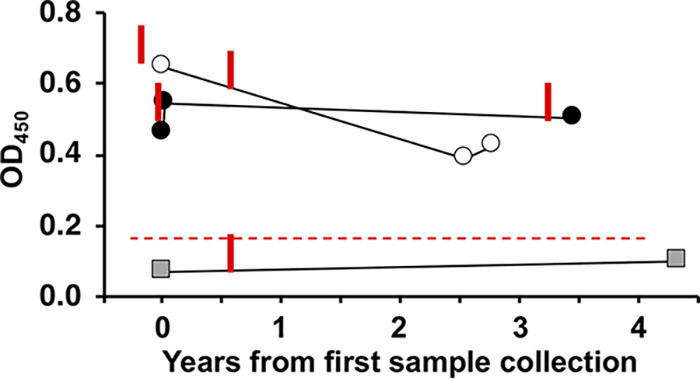
Longitudinal detection of anti-MABSC IgG. Plasma samples from two CF subjects with histories of MABSC infection (circles) and one subject with a transient MABSC infection (square) were collected at the indicated interval and anti-M. abscessus IgG was measured on plates coated with M. abscessus lysates. Positive cultures for each subject are indicated by the red bar touching the line. The red dashed line indicates the ROC cut-off (0.179) from Fig. 2A.

Samples for two subjects with a history of MABSC-positive cultures and one subject with a transient MABSC culture were available for longitudinal testing (Fig. 5). IgG levels were sustained over approximately 3 years for those with a history of MABSC infection, whereas the subject with a transient infection had low levels at both times, while having a known positive MABSC culture between these points. We previously reported sustained IgG and IgA in a separate study over the course of 5 years (8).

## DISCUSSION

Infection with MABSC organisms is a major concern in those with chronic lung disease, and notable in those with CF. The standard detection method in CF of sputum culture is becoming more challenging due to reduced sputum production during highly effective modulator therapy. In this context, assays less reliant on sputum production for the early and reliable detection of MABSC are important. Alternatives to determine infection status would also be useful in other chronic lung diseases were NTM infections are increasingly common, including chronic obstructive pulmonary disease and non-CF bronchiectasis (1, 2). In addition, extensive sputum decontamination impacts the sensitivity of NTM culture results compared to that for common CF pathogens (4–6). A blood-based assay to monitor disease status and impact of antimicrobial therapy would complement sputum culture results, or other tests developed in the future, such as targeted NTM sequencing from respiratory samples or urine biomarkers (24).

Previous studies of anti-MABSC plasma antibodies relied on detection of antigens found in all mycobacteria (19–22) and studies have shown the utility of proteinaceous extracts of M. abscessus and rPLC as antigens for detection of anti-M. abscessus IgG (23, 25). We reasoned that a complex antigen mixture would improve detection of anti-MABSC. The M. abscessus lysate used in this study contains most possible antigens, is simple to prepare, and demonstrated reproducibility. The ATCC strain used belongs to the worldwide dominant circulating clone in CF (26), and therefore, would be expected to be useful in detecting common strains, but may not capture specific antibodies produced against rough morphotype infections. The antigen enrichment strategy is beneficial in that the conditions promote simultaneous detection of all antibodies to nonsecreted antigens and the conditions for IgG detection (dilutions of 1:1,000 to 1:10,000) are far more dilute than other reported NTM ELISAs using single or fractionated preparations to reduce nonspecific binding (17, 20–23, 25).

The lysate assay performed best to detect IgG, which discriminated MABSC+ from MAC+, from subjects who had cleared MABSC, and from NTM-free subjects. Although less profound, MAC+ subjects were also distinguished from NTM-free individuals. The results indicate that antigens common to MABSC and MAC do not interfere with the sensitivity to detect MABSC infections. Sensitivity and specificity were similar, or improved, to those seen in other serodiagnosis studies (16–23, 25). No correlation of Ig levels to time of most recent positive culture was found, indicating that factors other than culture positivity were responsible for elevated Ig, including the ability of the host mount an effective humoral response or availability of mycobacterial antigens. IgA levels were also able to discriminate MABSC+ subjects from NTM-free subjects, but with a lower specificity. In contrast, IgM was not useful to determine NTM infection status. While some subjects had elevated plasma IgG and IgM levels perhaps indicating a more recent activation of anti-NTM B cells, the highest IgG expressors had the lowest IgM indicating effective early class switching and could be useful for classification of infection. Higher IgM may also reflect the lower specificity of IgM or production of IgM against ubiquitous environmental exposure. Overall, these data demonstrate the utility of detecting M. abscessus-specific IgG and IgA using this system.

The utility of IgG to distinguish MABSC+ subjects was confirmed in a blinded cohort of CF subjects. The elevated IgG signals in some NTM-free samples could reflect the heightened susceptibility of the CF population to environmental NTM exposure, a subsequent failure in innate immunity to control initial infections, and the need to initiate adaptive immunity, which then resulted in bacterial clearance in some subjects. In the case of false negative readings, the window of 3 years pre- and postculture positivity is expected to lead to an underestimate of assay sensitivity due to transient infections in some subjects. This window was selected based on clinical and genetic data suggesting that first positive culture lags time of infection by up to 3 years (7, 8) and the low sensitivity of NTM cultures.

Chronic infections are typically associated with class-switching (27–29). However, the major contribution of the IgG signal in this study appears to be IgG3 with only minor contributions from the other IgG subclasses. In contrast, IgG produced against Pseudomonas aeruginosa chronic infection in CF has been shown to include all four IgG subclasses (28–30). Specific IgG subclasses change with progression of infection (27–29), but there is strong evidence for an initial increase in IgG3, followed later in infection with IgG2, levels of which are correlated with poor lung function. Although IgG1 is the most abundant subclass in plasma, IgG3 is often the earliest class-switching IgG subclass to appear after infection (31, 32). In the IGH gene locus, which codes for all the Ig classes and subclasses, IgG3 is the first locus positioned downstream to that of IgM, the first Ig made in response to antigen before maturation to other classes. The predominance of IgG3 in chronic NTM infection suggests that further IgG maturation is restricted. Murine IgG3 restriction has been previously demonstrated for T cell-independent type 2 polysaccharide antigen response (33). Detection of antigen-specific T cells have also been identified as a possible diagnostic tool (34). The peripheral response during lung infection is poorly understood and requires additional study. The lack of abundant IgG2, which has enhanced specificity to carbohydrate antigens is surprising considering abundant carbohydrate production by mycobacteria, although IgG3 can also be directed against carbohydrates. IgG4 is a rare subclass and often appears during chronic, noninfectious antigen exposure such as in allergy (35). Therefore, in addition to showing the utility of measuring anti-MABSC antibodies as a marker of NTM infection, these studies have identified details of the humoral immunological response during chronic infections.

Antibodies are detected to both water- and organic-soluble antigens as both cell lysate fractions detected anti-M. abscessus IgG similarly. This is consistent with the findings that anti-NTM antibodies to both protein and lipid antigens have been identified (17, 19, 21–23, 25, 36), and the high level of lipid in NTM. The contribution of lipoproteins and other complex molecules to these results is unclear. An interesting observation is that the subjects with the highest readings in Fig. 2A and those in the Fig. 3A and B are not the same individuals. This could be due to differences in antibody specificity or loss of antigens due to incomplete extraction of the fractions.

Our results suggest that this assay could be generalized to detect anti-MAC antibodies. We made M. avium lysates for use as antigen and performed an ELISA with the training plasma samples. Two surprising findings from these studies were the lack of detection of specific antibodies in the plasma of MAC+ subjects and the ability of M. avium lysates to detect IgG from MABSC+ subjects with high specificity (Fig. S6). This finding is reminiscent of recently published results showing low response of MAC+ subjects to an M. avium specific antigen (37). However, based on previous studies (17, 19–21), MAC+ subjects do produce specific antibodies. The data may reflect the possibility that MABSC+ subjects make IgG to antigens that are common to NTM, but MAC+ subjects may mount a humoral immune response only to antigens that are lost or not expressed during NTM culture or preparation of NTM lysates.

Limitations to this study exist. While the antigen mixture used in this study effectively detected specific antibodies, the identification of defined MABSC antigens could improve the specificity of this assay. Recent studies have used rPLC and a TLR2-activating protein fraction to detect MABSC infections with high specificity and sensitivity (23). Directed strategies could include the use of MABSC+ plasma to affinity purify specific antigens. The use of cell lysates may prove useful in screening for specific antigens through genetic targeting of candidates. These results are tempered by the higher variability in the test cohort. Larger cohorts are needed to test the clinical utility of this assay, and explorations of distinct antigens may improve specificity. Similarly, the specific detection of MABSC+ subjects could be due to the difference in collection time postpositive culture between MABSC+ and MAC+ subjects; however, this difference was not significant and we did not detect a correlation between collection time and IgG signal (Fig. S1 and S5). Combinations of biomarkers may more effectively detect infection. Poor detection of IgG1, IgG2, and IgG4 against M. abscessus could be due to the low sensitivity of the detection antibodies; however, two different IgG1 and IgG4 antibodies were used with similar results. Additionally, IgG subclass maturation to specific antigens could have occurred, but the signal could have been overcome by the breadth of antibodies produced to diverse antigens, which are presented at their natural levels instead of the concentrated levels used when recombinant or isolated components are used. Finally, while useful for diagnostic purposes, the role of antibodies in the host response to infection is not well understood and not addressed. The discovery of a distinct immunoglobulin profile, consisting primarily of IgG3 and IgA, may lead to a better understanding of host mechanisms for NTM control, and provide a rationale for studying the B cell response and functional properties of anti-NTM antibodies.

## MATERIALS AND METHODS

### Study populations and clinical evaluation.

PwCF were recruited from the Adult CF Program at National Jewish Health and the Pediatric CF Center at Colorado Children’s Hospital from 2014 to 2019. Subjects were recruited from both clinical and hospital settings. In the training cohort, patients were recruited based on known or suspected NTM culture status, as described previously (24). Healthy subjects were recruited from the general population and were screened to exclude recent illness. Studies were Institutional Review Board approved and informed consent was obtained before sample collection.

### Serum and plasma processing.

Blood was collected and isolated plasma was stored at −20°C or −80°C.

### ELISA.

Heat-killed M. abscessus (ATCC 19977) cultures were grown for 3 days in 7H9 with 10% ADC supplement to an OD_600_ ~2.0 (38). Cultures were washed in water and probe-sonicated for 10 s at approximately 4 W. Pelleted cells were resuspended in 7 mL 0.05 M bicarbonate buffer, pH 9.6, incubated at 65°C for 60 min, and the resulting suspension sonicated for 20 s. One-hundred ng protein in 0.05 M carbonate buffer, pH 9.6, was dried overnight in wells of a 96-well plate, blocked with 0.5% BSA/phosphate buffered saline (PBS), and incubated overnight at 4°C with plasma sample dilutions in 0.1% BSA/PBS. Sample dilutions used were: IgG, 1:10,000; IgG1, 1:2,500; other IgG subclasses, 1:1,000; IgA, 1:2,500; and IgM, 1:2,500. Wells were washed in 0.1% BSA/PBS and bound antibody detected with HRP-conjugated anti-human antibodies (1:10,000) specific for IgG (abcam, ab97175), IgM (Southern Biotech, 2020-05), IgA (Southern Biotech, 2050-05), IgG1 (Southern Biotech, clone HP6001), IgG2 (Invitrogen, clone HP6014), IgG3 (Invitrogen, clone HP6047), and IgG4 (Invitrogen, clone HP6025) followed by incubation with TMD (SureBlue) for 15 min and stop solution. Positive OD_450_ cut-offs were determined from the point of ROC curves closest to the upper left corner of the graph. Results were similar if the ROC curve analysis or 2 SD cut-offs were used (not shown).

### Statistics.

Culture results were used to classify NTM status, and the specificity and sensitivity for each assay were determined based on assay cut-offs derived from receiver-operator characteristic curves (http://www.rad.jhmi.edu/jeng/javarad/roc/JROCFITi.html). Data are plotted for each individual subjects and includes the mean +/– the standard deviation. Differences between groups were determined by *t* test. Linear regressions were performed in Excel. The coefficient of variation was calculated by dividing the standard deviation to the mean.
