# Supplementary material for: Specificity of Immunoglobulin Response to Nontuberculous Mycobacteria Infection in People with Cystic Fibrosis
Source: Microbiol Spectr. 2022 Jul 6;10(4):e01874-22. doi: 10.1128/spectrum.01874-22 (PMC9430546; doi:10.1128/spectrum.01874-22)
Supplement: Supplemental file 1 — Figures S1-S6 and Table S1. Download spectrum.01874-22-s0001.pdf, PDF file, 0.5 MB [file spectrum.01874-22-s0001.pdf]

Supplemental Materials

Supplementary Methods.

Hierachical clustering and heat map. Data were analyzed in Morpheus (https://software.broadinstitute.org/morpheus) using 1 minus Pearson correlation and average linkage.

Organic extraction of lysates. For assays designed to distinguish recognition of aqueous and organic soluble antigens, approximately 0.5 g of *M. abscessus* lysate in carbonate buffer was extracted with twice the original culture volume of $CHCl_3$/MeOH (2:1) with vigorous mixing for 1 min, settling at room temperature for 1 hr, followed by centrifugation for 2 min at 20,000 g. Organic and aqueous (including the interface) phases were cleared by centrifugation at 20,000 x g, 10 min at 22°C. The aqueous and organic phases were back extracted with 500 μl $CHCl_3$ and 200 μl H_2O, respectively, and normalized to volume. Plates were coated with 100 ng protein from the aqueous fraction and an equal volume from the organic fraction.

Anti-MAC ELISA. Assay. Heat-killed *M. avium* (CF002 from the Colorado NTM Research Development Program) cultures were probe-sonicated, and 100 ng protein was dried overnight in wells of a 96-well plate. Plates were blocked with 0.5% BSA/PBS and incubated overnight at 4°C with plasma sample dilutions in 0.1% BSA/PBS. Samples were diluted 1:10,000. Wells were washed in 0.1% BSA/PBS and bound antibody detected with HRP-conjugated goat anti-human IgG (abcam, ab97175). Positive OD_{450} cut-offs were determined as 2 standard deviation higher than the average from NTM-free CF subjects as the AUROC was 0.31.

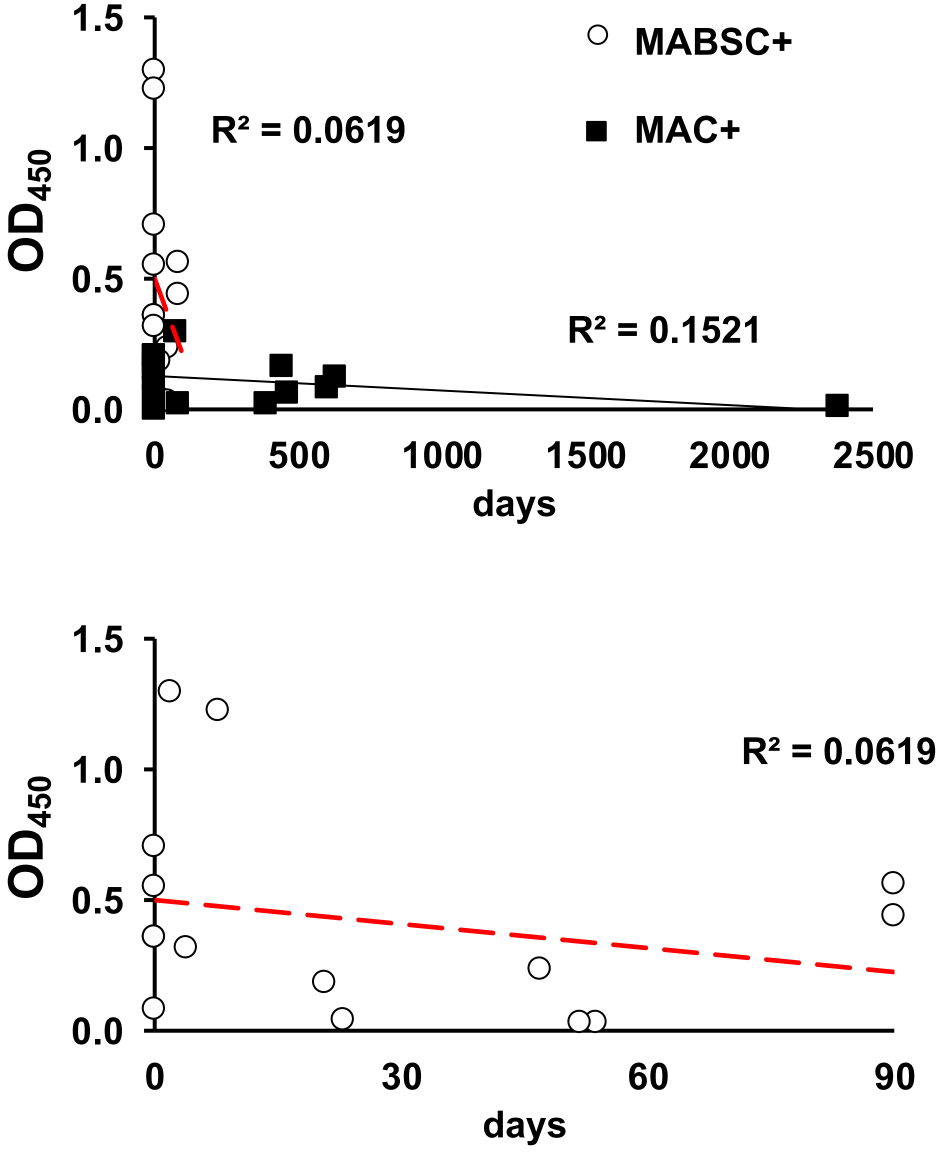

Supplementary Figure S1. The level of IgG signal is not associated with the time from last positive culture. *Upper panel*, The OD_{450} readings as a function of the time of sample collection from the last positive culture for MABSC (*open circles*) and MAC (*closed squares*). Regression lines and R2 are shown for MABSC (Red dashed line) and MAC (solid line). The P-vaules for MABSC and MAC were 0.37 and 0.16, respectively. The *lower panel* isolates the MABSC samples.

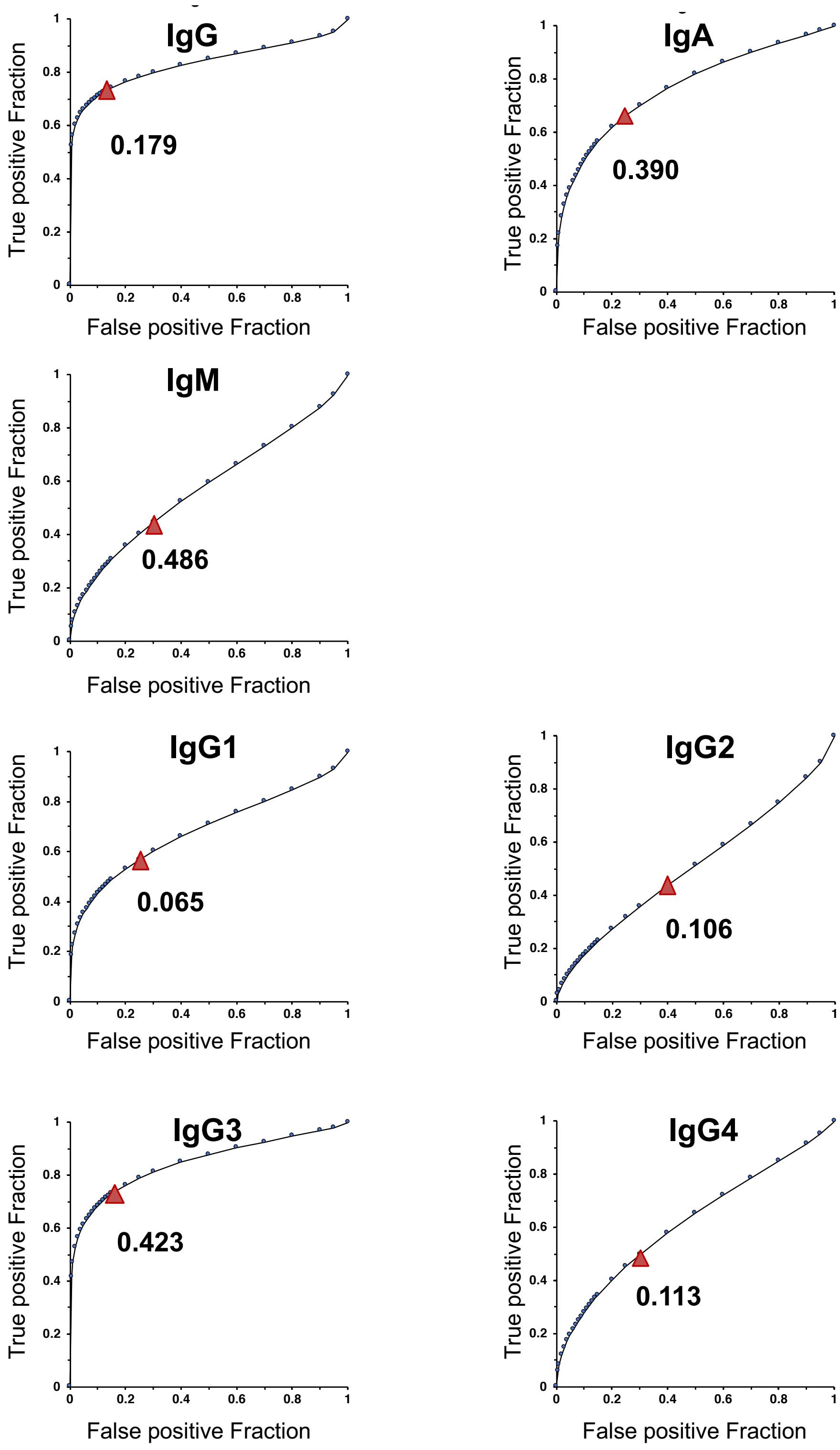

Supplementary Figure S2. Receiver-operator characteristic (ROC) curves for each of the antibodies tested. The red triangle depicts the estimated point on the ROC curve to derive the OD_{450} cut-off value, which is indicated on each graph.

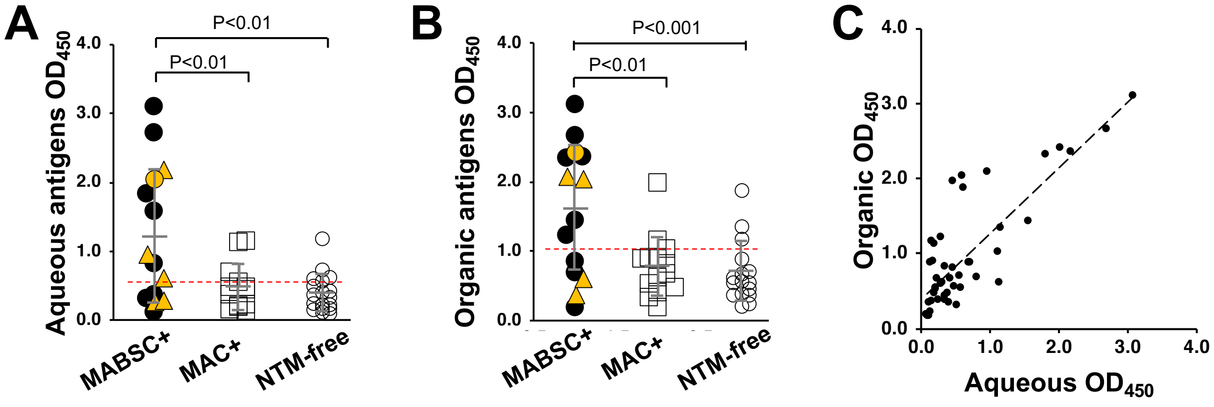

Supplementary Figure S3. Detection of MABSC IgG against antigens in aqueous and organic fractions. Detection of IgG binding to plates coated with (**A**) aqueous fraction, and (**B**) organic fraction of *M. abscessus* lysate. (**C**) Correlation of signals from aqueous- and organic-coated plates. Red dashed line represents the cut-off for detection determined from the ROC curve. Cut-off values were 0.535 (aqueous) and 1.013 (organic). *M. abscessus* positive subjects (closed circles), *M. massiliense* (orange triangles), and *M. bolletii* (orange circle); MAC+ (open square); NTM-free (open circle); grey bars indicate mean +/- SD.

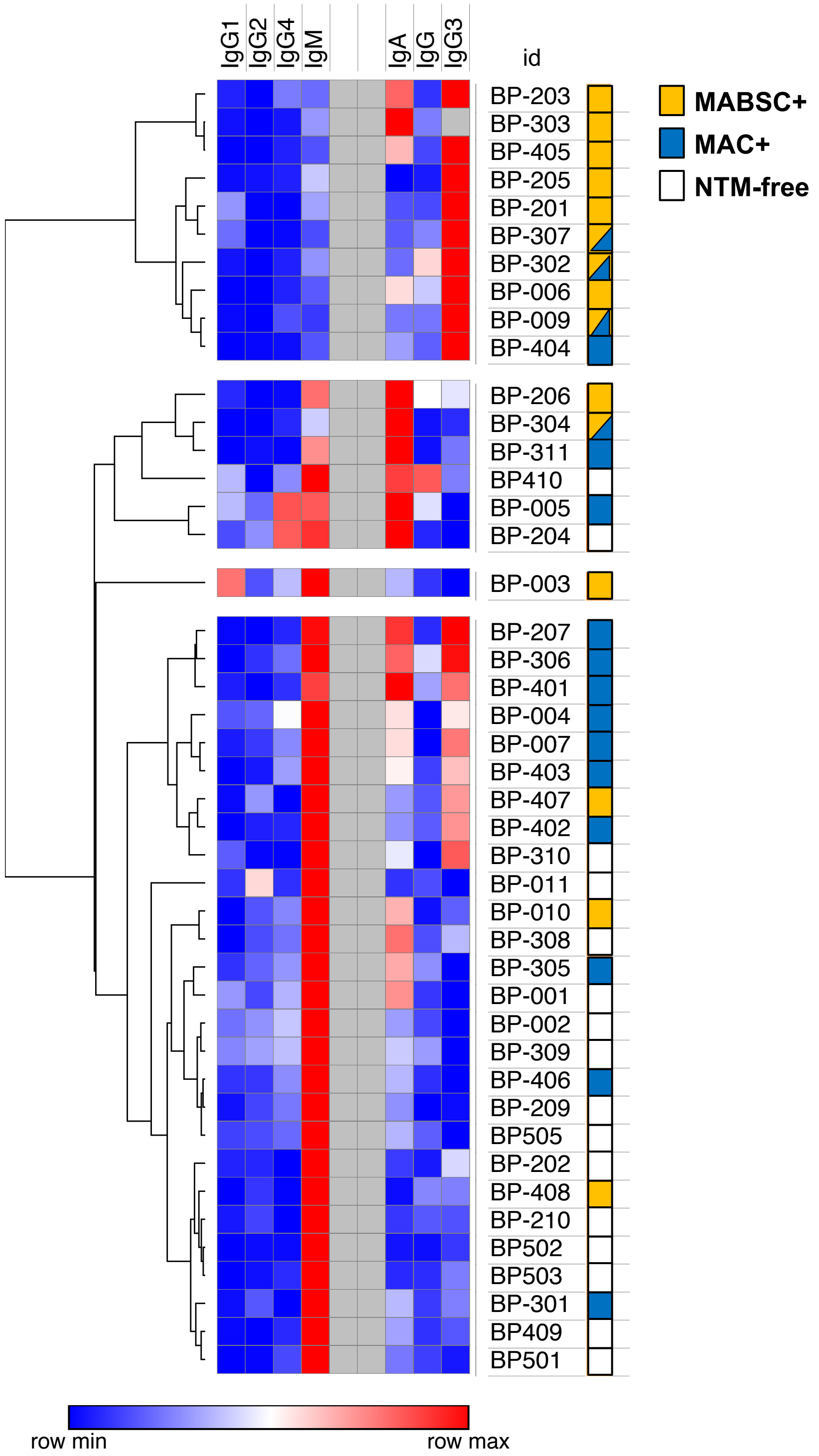

Supplementary Figure S4. Heat map of anti-*M. abscessus* antibody levels. Data were analyzed by hierarchical clustering. Culture status of each subject is provided at the right. Orange, MABSC+; blue, MAC+; white, NTM-free; subjects with cocultures of MABSC and MAC are depicted in both orange and blue.

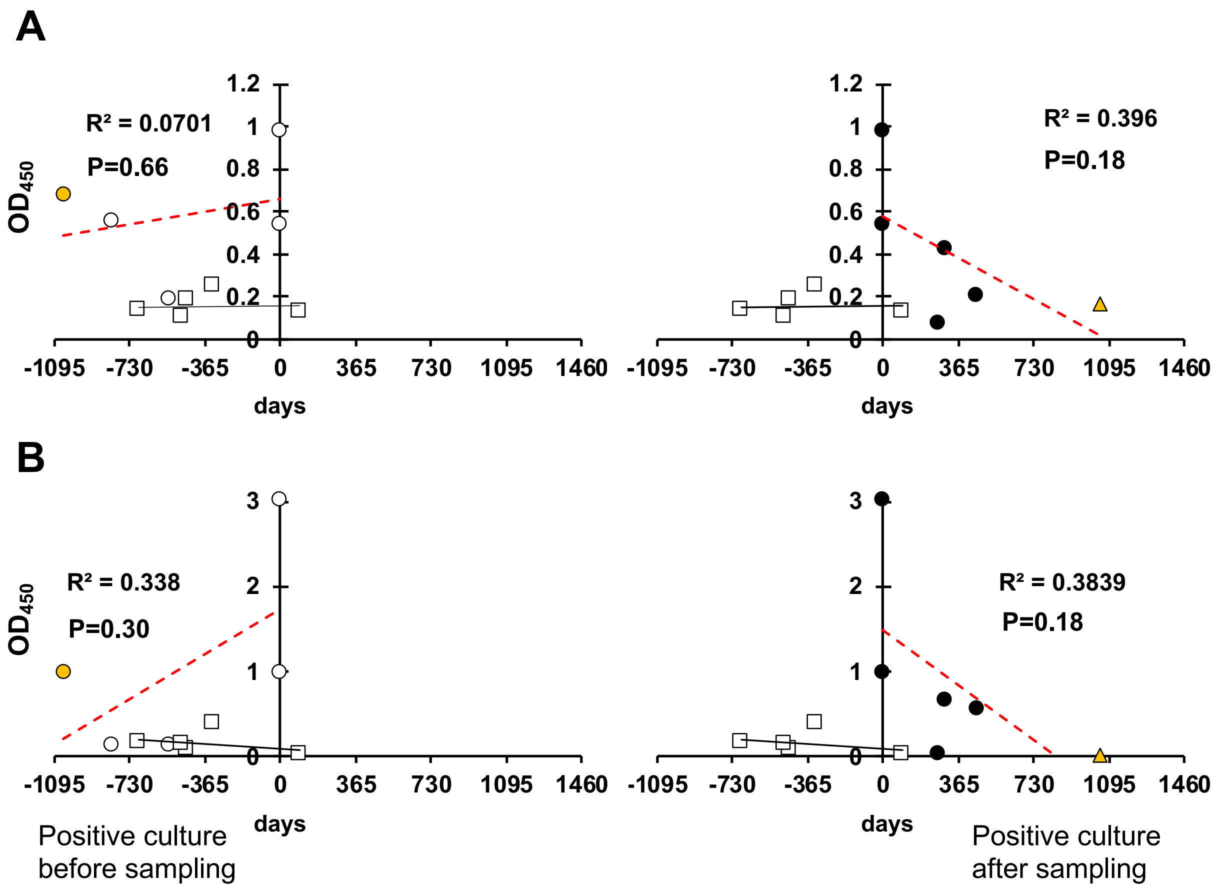

Supplementary Figure S5. The level of IgG signal is not associated with the time from last positive culture in the validation cohort. The level of (**A**) IgG, and (**B**) IgG3 is not associated with the time from last positive culture, when that last positive culture was before or after sample collection. OD_{450} readings when subjects cultured positive before (*left panels*) and after (*right panels*) sample collection at day 0. Symbols represent M. abscessus (*circles*), *M. bolletii* (*orange circle*), *M. massiliense* (*orange triangle*) and MAC (*squares*; shown in all graphs for reference). Regression lines (*Red dashed line*) and R^2 are shown for MABSC+. Regression lines only are shown for MAC (*solid line*); R^2 for MAC+ IgG and IgG3 were 0.003 and 0.15, respectively. The P-vaules for the MABSC+ are shown.

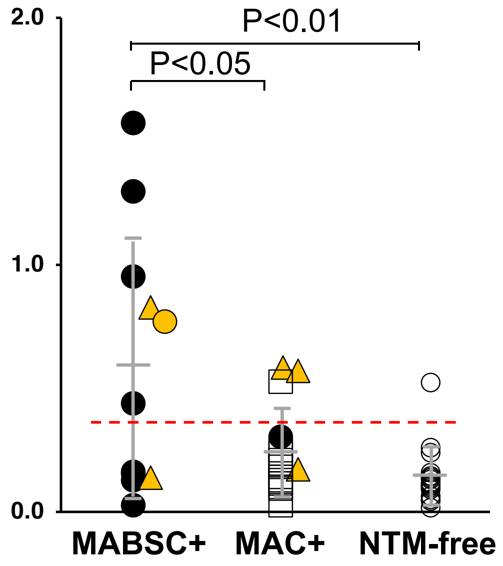

Supplementary Figure S6. Plasma antibodies from MABSC+ subjects recognize *M. avium* antigens. Detection of IgG binding to wells coated with *M. avium* lysates. MABSC+ (closed circles), MAC+ (open squares) and NTM-free subjects' (open circles) plasma samples were tested for binding to *M. avium* antigens. Samples were identical to those used in Fig 2. Red dashed line represents the cut-off using two standard deviations from the mean of the NTM-free subjects. The specificity and sensitivity for detecting MAC-positive (45% and 18%, respectively) and MABSC-positive (90% and 50%) subjects with *M. avium*-coated plates inversely correlated to infection status. Two of the MAC+ samples over the cut-off were co-infected with MABSC (closed symbols). *M. abscessus* positive subjects (closed circles), *M. massiliense* (orange triangles), and *M. bolletii* (orange circle); MAC+ (open square); NTM-free (open circle).

Table S1. Descriptive statistics for detection of MABSC+ by aqueous and organic fractions of *M. abscessus*.

	IgG aqueous	IgG organic
Sensitivity (%)	64.3	64.3
Specificity (%)	71.4	78.6
PPV [1] (%)	69.2	75.0
NPV [2] (%)	66.7	68.8
AUROC [3]	0.73	0.77

[1] PPV, positive predictive value; [2] NPV, negative predictive value; [3] AUROC, Area-under-the-ROC curve.
